# Takes more than two to tango: Intrahousehold food system agency and its intricacies in South Africa

**DOI:** 10.1016/j.heliyon.2023.e21770

**Published:** 2023-11-10

**Authors:** Saul Ngarava

**Affiliations:** Copernicus Institute of Sustainable Development, Vening Meinesz Building A, 8a Princeton Avenue, 3584 CB, Utrecht, Netherlands

**Keywords:** Agency, Capability approach, Intrahousehold, Monte Carlo simulation, Poisson regression, Propensity score matching, South Africa

## Abstract

Intra-household food system agency is affected by individual physiology, environmental diversity, variations in social conditions, relational perspectives and distribution within families, resulting in differing capabilities and functionings which have a bearing on welfare. The study used the capability approach to assess intrahousehold food system agency, its determinants and welfare impact, taking a cross-sectional survey of 1184 households from three heterogenous study sites in South Africa. Poisson count regression, Propensity Score Matching and Monte Carlo Simulations were used in the study. Duration of stay within the community, tenure, employment status and household size were significant in the number of intrahousehold food system decisions made by female heads and none-nuclear family members, with however overall reduction in number of decisions made. Yet, when female heads showed agency in agricultural production and food expenditure, food security improved by 5 % and 6 %, respectively. On the other hand, it is reduced by between 4 % and 5 % for none-nuclear family members. Duration of stay in the community and age of household head had the highest contribution to agency variation. In conclusion, there are varied intrahousehold food system decisions made by various members of the households, resulting in varying degrees of food security. In addition, variations in the socio-economic factors of the households result in varied agency, especially for female household heads and other none-nuclear family members. Female-head intrahousehold food system agency should be promoted in lieu of their positive impact on food security, and their reduced decision-making capacity in hindsight.

## Highlights

• Differentials in agency, utilities and welfare gains based on intrahousehold decision-maker.
• Female head agency increases food security, external family agency decreases it.
• Overall, intrahousehold food system decision making reduces for female heads while increasing for external family members.

## 1 Introduction

An agent is an individual acting to bring about change, not only on their own behalf but for others [1,2]. Agency is achieved through power, which can be augmented by converging individual agency to collective agency [1]. A household consists of a group of people living together, pooling their resources and eating at least one daily meal together [3,4]. Thus, even though a household strives for collective agency due to the fact that it is a group, it can be shaped by the intrahousehold power dynamics trying to achieve individual agency. However, there are differing preferences for individuals within collective households, affecting resource allocation and distribution, and ultimately welfare [5]. Various approaches have been used to understand intrahousehold decision-making. These stem from the unitary model which assumes that the household acts as a single decision-maker, pooling all resources together, to the collective household models indicating that households reach efficient outcomes from individual-level factors affecting these outcomes (Pareto efficiency). However, these studies have not been conclusive in intrahousehold decision-making, with some finding antagonistic Pareto inefficiencies [6].

Various studies have been conducted which have focused on intra-household agency. In Tanzania, Anderson [7] highlighted that wives had authority over farming and household decisions with variations in the allocation of authority between men and women and amongst women themselves. Furthermore, authority was bolstered by increased educational levels, age, health and employment status. The study further identified that the particular farming or household decisions made were also significant in establishing authority which was also significant in restricting intra-household accord [7]. Kafle [8] also found that in Tanzania, there was an increase in joint decision-making, having welfare gains through increased cooperation within households. The studies were however devoid of incorporating other nuclear and non-nuclear family members in the decision-making dynamics. This was also the same limitation in the study by Verschoor et al. [9] in India, Ethiopia and Nigeria. The study found that when spouses pursue different productive activities, there is inefficiency in intra-household decision making. Furthermore, female authority reduced efficiency. Simiyu [10] and Shibata et al. [11] found that in Uganda, there were differences in food production and consumption activities, with women exercising greater authority over subsistence food production and consumption, while men focused on income-generating food or cash crop production activities. This was supported by Ashraf [12] in the Philippines, who found male household heads having affinity in private financial investments which are self-benefiting and high self-utility when investments are joint. Furthermore, Mohammed et al. [13] found that there was higher food security for households that engage in joint decision-making in Ghana. This is premised on how joint decision-making allows negotiation and reconciliation of preferences in resource allocation. In Colombia and Nicaragua, Godek and Garcia [14] discovered that gender transformative decision-making is restricted by traditional gender norms and perceptions on food decisions, information asymmetries in gender equality and human rights and how they are shaped in the home. Food security welfare outcomes were shown to increase when intra-household decision-making was dominated by females in Ethiopia [15]. Ngenzebuke et al. [16] factored in the influence of kinship in a study carried out in Burundi, indicating that female authority was increased when the immediate family was rich than the husband's, with issues such as proximity to extended family, relative wealth and family size also being significant. In South Africa, Guvuriro [17] highlighted that increased authority by females increases expenditure on family-type goods. Despite a plethora of these studies, they appear to focus on the household heads, neglecting other nuclear and non-nuclear family members. Furthermore, the impact of such agency on welfare outcomes has not been adequately evaluated. The pertinent question that the study tries to answer relates to established agency in intrahousehold food system decision-making, who “tangos” in this arena and how this affects welfare outcomes of food security.

Bernard et al. [18] identified 5 typologies of households, which may overlap, depending on how decisions are made. These include “unitary” model where all decision are made by one single person; “contribution” model based on individual contributions, whether in terms of factors of production; “separate spheres” model where there are separate domains to make decisions leading to specialization; “norms” model, where the community determines the decision-maker; and “most informed” model where the decision-maker is more knowledgeable [18]. However, most intrahousehold decision-making literature focusses on husband and wife, taking the unitary approach, where the decisions are jointly made, neglecting none-nuclear family [19]. Furthermore, intrahousehold food system research has focused on consumption and distribution at the expense of production [[20], [21], [22]]. Lesser still, a few have focused on agency. According to Harris-Fry et al. [23] literature on intrahousehold food decision-making was speculative, theoretical and anecdotal, mainly focusing on bargaining power, itself difficult to measure [18]. De Backer, Holvoet and Milanzi [19] acknowledges that there are changes in practices and circumstances resulting in dynamics in intrahousehold agency. Thus, findings from previous literature are never static, requiring further inquiring which incorporates this variability. Even though Bernard et al. [18] indicated there is literature on intrahousehold power dynamics, there is less attention on how decisions made affect household welfare. The objective of the study was to assess intrahousehold food system agency, its determinants and its impact on food security, taking 3 heterogenous cases in South Africa.

The study was beneficial in providing a referral literature that incorporates other non-nuclear family members in intra-household food system agency. The study also considered non-household heads in intra-household food system agency. This is significant in lieu of hidden food insecurities and agency that have been identified in literature to exist at the individual level [24,25]. Households have differing needs, wants and utilities. This can have profound effects on micro-individual agency and welfare outcomes. The study was also essential in providing literature on intra-household food system agency and welfare outcomes in South Africa. Besides the limited literature, the country has high levels of socially constructed inequalities based on gender roles, age, ethnicity and race which reflects economically through income [26,27]. The study is also significant in informing individual level food security and indigent support policy. South Africa has been deemed a micro-level food insecure country even though it exhibits macro-level food security [28]. The micro-measure has been relegated to the household level neglecting individual food security outcomes. The study not only brings this to the fore, but it also further establishes who has the decision-making capacity. This is significant in crafting targeted policies such as food security, indigent support or even other social protection policies such as child grant and old age grant. South Africa's school feeding scheme through the National School Nutrition Programme (NSNP) [29] can also benefit from insights which take into account the intra-household welfare outcomes of individual agency. This is through informed targeting to alleviate short term hunger of school going children who suffer from masked food insecurities hidden under the guise of household security. The food and agriculture private sector can find the findings also appealing. The findings can be used to inform their products in terms of market segmentation and targeting especially when they have the purview of the intra-household food system decision-makers. This will have an effect on their bottom line.

## 2 Methodology

### 2.1 Study site

**Fig. 1:**
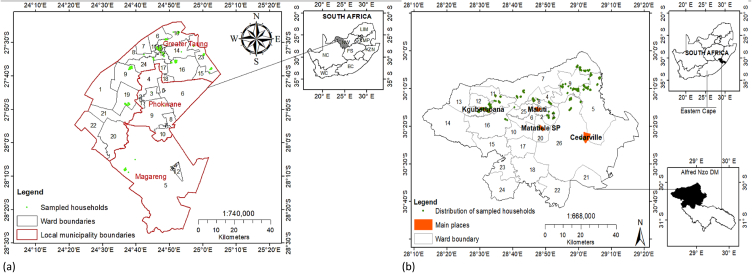
Study area showing (a) Greater Taung and Magareng Local Municipalities and (b) Matatiele Local Municipality.

The study was carried out in 3 local municipal areas of South Africa, namely Matatiele (Eastern Cape), Magareng (Northern Cape) and Greater Taung (North West) (Fig. 1 (a,b)). The characteristics of the study sites are comparably shown in Table 1. Matatiele Local Municipality (MatLM) is characterized by a predominately rural and struggling subsistence sector in the former Transkei and Ciskei which is surrounded by highly developed farm lands [30,31]. It sits on 4356.9 km^2^, accommodating a population of 219 448 with 48 % being under the age of 18 and a 40 % employment rate, as well as 22.1 % of the household having a monthly income less than R2 000 [32,33]. Close to 18.7 % of the people in MatLM were in poverty in 2016, with food insecurity being one of the threats to economic development compromised by an underdeveloped agricultural sector and the insignificant contribution of agriculture to the local economy, which is mainly dominated by livestock production [31,34]. In the Northern Cape, Ward 5 in Magareng Local Municipality (MagLM) was used in the study targeting land restitution beneficiaries. According to Wazimap [35] Ward 5 in MagLM has a population of 3139 in 1075 households. Nearly 60 % of the population is between 18 and 64, with 51 % being male. Tswana and Afrikaans ethnic groups constitute 45 % and 44 % of the population, respectively. Close to 27.2 % of the households in Ward 5 are female headed, with average annual household incomes of R29 400. There is a 51 % unemployment rate in the Ward, where 37.2 % of the population has completed secondary and higher education. MagLM is comprised of urban, villages and farm nodes, with Ward 5 consisting of Warrenton town and surrounding rural areas [36,37]. Dominant agricultural activities in MagLM are livestock farming [38]. Grater Taung Local Municipality (GTLM) has a population of 167 827 predominately Setswana speaking on 5647 km^2^ [39]. The local municipality has 46 168 households with a 50 % unemployment rate, with those employed earning an average of R15 000 annually. GTLM is predominately rural, with 106 villages and agriculture being one of the main economic activities, dominated by poultry and livestock production [40,41]. The municipality has a poverty rate of 76.6 % people and high food insecurity [42,43].

**Table 1:** Characteristics of the study sites.

	Matatiele	Magareng	Greater Taung	South Africa
Population	219 448	24 060	167 827	55 653 654
Population under 18 (%)	48	18.38	44.0	38.0
Households	56 868	6970	46 168	16 923 307
Area (km^2^)	4 3 56.9	1548.7	5647	1 229 341.5
Employment rate (%)	40	24.4	50	38.9
Monthly income less than R 2000 (%)	22.1	26.0	27.0	19.0

### 2.2 Conceptual framework

The study utilized a capability approach, proposed by Sen [44,45], which is an interdisciplinary evaluative framework focusing on individual's ability to exercise agency (i.e. ability to act) as suggested by Nussbaum [46]and Robeyns [47]. It highlights that different people require different types of capability input to attain the same well-being [48]. It indicates the economic, social, personal, cultural and institutional factors allowing opportunity for individuals to do and to be what they consider valuable for their fulfilment, referred to as socially constituted generative capacities of dispositions by Otto and Ziegler [31] and Bourdieu [49]. According to Well [50], capability approach focusses on individual quality of life, which is assessed in term of functionings and capability. This is because well-being evaluation should consider what people can actually be and do, irrespective of their wealth or mental reactions. Capability are a set of valuable functionings an individual has effective access to, i.e. the effective freedom to choose between different functioning combinations for an individual, whereas functions are a state of being and doing, e.g. a state of wellbeing, distinguished from the commodities used to achieve this wellbeing [50].

**Fig. 2:**
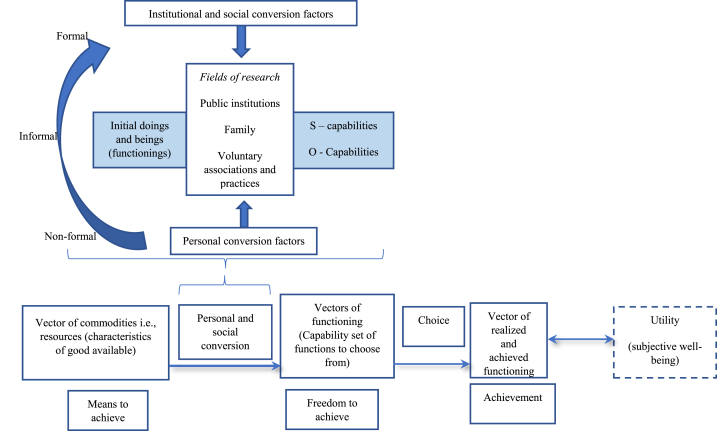
The Capability Approach conceptual framework.

**Source:** Adapted from Sen [27], Otto and Ziegler [31] and Well [33].

As shown in Fig. 2 there are various factors that influence how a person converts capability inputs into capabilities, namely personal conversion factors (e.g. competences, literacy, physical condition etc.), socio-structural and cultural conversion factors (e.g. discriminatory practices, power relations and hierarchies, gender roles, social or religious norms etc.) and institutional conversion factors (e.g. collective provision, educational arrangements, welfare, etc.) Otto and Ziegler [48]. Resources are inputs whose value depends on the ability of individuals to convert them into valuable functionings depending on their personal physiology, social norms, and physical environment. Personal conversion determines how individuals convert arrangements, infrastructure, commodities and characteristics into functionings (full range of activities including caring, re-productive, productive etc.). Utility is both a functioning and output. As an output, people chose what to do, affecting their subjective well-being. However, sense of satisfaction as a subjective-wellbeing is a valuable functioning [50].

Otto and Ziegler [48] managed to distinguish between O (option and opportunity) and S (relating to skill and substantive) capabilities (Fig. 2). The S capabilities refer to the skills, abilities and attitudes while O capabilities combine individual agency (e.g., cognitive abilities, psychological factors, etc.) and social agency (structure and institutions, etc.). O capabilities tend to focus on “what an individual is able to do” and “which opportunities are open for him or her” [48].

The capability approach can be used to assess relationships between people and commodities e.g. individual physiology (age, gender, disability etc.), local environment diversities (environmental pollution, climate change, epidemiology etc.), social condition variations (community relations, such as class and ethnic divisions, security, education etc.), differences in relational perspectives (conventions and customs) and distribution within family (rules determining food and health allocation between males, female, adults and children) [50]. Thus, from the current study (which focusses on the latter), individuals capability set (within households) may include different functioning relating to various food system activities such crop and vegetable production, livestock production, cooking and food expenditure. Individuals may choose to partake in any of these activities. Intrahousehold individuals tend to acquire both utility from the food system activity they have agency on and the satisfaction obtainable, and thus overall improving their (and the collective) well-being.

## 3 Study design

Multi-stage purposive sampling was used on the cross-sectional survey that was carried out in the study. Purposive sampling was used in selecting the provinces, municipalities, wards, villages and households. Household selection was based on their availability and was not informed by any probability measures. The discriminatory selecting criteria and sampling frame was informed by beneficiaries of land restitution [36,37], the Matatiele Spatial Development Framework Review [51], Integrated Development Plans [52,53] and traditional leadership informants focusing on poor rural households. Yamane [54] method (Eq. (1)) was used to calculate a proportional sample size.

(1) $$n=\frac{N}{1+N{\left(e\right)}^{2}}$$

where n was the sample size, N is the population, which was 35 580 households (from 16 purposively selected wards in the 3 study sites), and e was the degree of accuracy, which was 95 % in the study. The sample size was calculated as follows (Eqs. (2), (3))):

(2) $$n=\frac{35580}{1+35580{\left(0.05\right)}^{2}}$$

(3) n=396

The study ended up using a sample of 1184 households from the 3 study sites with distribution shown in Table 2.

**Table 2:** Sample size.

								Ward									Total
**Municipality**	**1**	**3**	**4**	**5**	**7**	**8**	**9**	**11**	**12**	**13**	**14**	**16**	**19**	**21**	**23**	**26**	**16**
Matatiele		38	50	71	77	43	78	80	55				2			55	549
Greater Taung	65						80	52	72	75	1	59	73	1	78		556
Magareng				79													79
Total	65	38	50	150	77	43	158	132	127	75	1	59	75	1	78	55	1184

### 3.1 Analytical framework

The study utilized a three-stage integrated process in data analysis. The first stage involved assessing the number of household food system decisions made and their determinants by the various agents. The next stage ascertained how the assigned decision-makers impacts various food system decisions pertaining to crop and vegetable production, livestock production and food processing (cooking) within the households. The last stage simulated how different conditions can affect food system decision-making in the households.

i. Poisson count regression model

The Poisson count regression model as used by Mukarumbwa et al. [55] was used to assess the determinants of intrahousehold agency in food system decision-making. The Poisson probability density function is shown in Eq. (4),

(4) $$f\left(Y_{i}|X_{i}\right)=P\left(Y_{i}=y_{i}\right)=\frac{e^{\gamma }\gamma^{\gamma }}{Y!}=y=0,1,2,...$$

where $y_{i}$ is the number of household food system decisions made, $X_{i}$ are the determinants of the number of household food systems decisions. γ is the mean parameter of this distribution, defined by Eq. (5),

(5) $$E\left[Y_{i}|X_{i}\right]=\gamma_{i}=\exp\left(x_{i}^{\prime }\beta \right)$$

where $\exp\left(x_{i}^{\prime }\beta \right)$ is the incidence rate ratio for one unit change in $X_{i}$, ceteris paribus. β is estimated using the maximum likelihood procedure from the function in Eq. (6).

(6) $$\ln\left(\beta \right)=\ln\left[\frac{e^{\gamma }\gamma^{\gamma }}{Y!}\right]=-\gamma \gamma +Y_{i}\;\ln\left(\gamma \right)-\ln\left(Y_{i}!\right)=\exp\left(x_{i}^{\prime }\beta \right)+Y_{i}\left(x_{i}^{\prime }\beta \right)-\ln\left(Y_{i}!\right)$$

ii. Propensity Score Matching

Propensity Score Matching (PSM) was used to determine the impact of agency on intrahousehold food system decision making. For a household p, (where p=1…P and P denotes the population of households), the impact evaluation separated the impact of a member of the household being a decision-maker (i.e. male household-head, female household-head, son, daughter or none-nuclear family member) ($D_{p}=1$) on a certain outcome $Y_{p}\left(D_{p}\right)$ [number of household food system decisions made i.e. crop, vegetable, livestock production, cooking and food expenditure as well as Household Food In-access Scale (HFIAS)] from what would happen had that family member had not made that decision ($D_{p}=0$), the counterfactual (Eq. (7)). This is the difference between the outcome of agency by one intrahousehold decision-maker for household p and the counterfactual potential before/without that agency on intrahousehold food systems.

(7) $$\omega_{p}=Y_{p}\left(1\right)-Y_{p}\left(0\right)$$

The impact $\omega_{p}$ cannot be observed since a household family member is either a decision-maker or is not, but never both. The next stage was to ascertain the average treatment effect of the treated (ATET) (Eq. (8)):

(8) $$\omega_{ATET}=E\left[\omega |D=1\right]=E\left[Y\left(1\right)|D=1\right]-E\left[Y\left(0\right)|D=1\right]$$

The resulting PSM estimator for ATET was generalized as (Eq. (9)):

(9) $$\omega_{ATET}^{PSM}=E_{\mathrm{Pr}\left(X\right)|D=1}\left\{E\left[Y\left(1\right)|D=1,\mathrm{Pr}\left(X\right)\right]-E\left[Y\left(0\right)|D=0,\mathrm{Pr}\left(X\right)\right]\right\}$$

In the PSM, a Probit model was used with variables in Table 3.

**Table 3:** Variables used in the PSM.

Variable	Explanation	Type of measurement	Expected sign
Outcome variable			
Agency in intrahousehold food systems	Number of intrahousehold food system decisions made (i.e., crop and vegetable production + livestock production + cooking + food expenditure)	Count: 0≤n≤4	
HFIAS Index	Household Food In-access Scale indexed between 0 and 1 through Min-Max Normalization	Fractional: 0≤n≤1	
**Treatment variable**			
Y	Decision-maker (for each food system activity i.e., crop and vegetable production, livestock production, cooking, food expenditure)	Nominal: 0-(Male household head; or Female household head; or Son; or Daughter; or Other none-nuclear family member), 1-Otherwise	
**Independent variable**			
AGE	Age of household head	Nominal: 0 - <40 years, 1 - >40 years	+
EDU	Educational level of household head	Nominal: 0-None, 1- Otherwise	–
TEN	Tenure	Nominal: 0-Own, 1- Otherwise	–
EMPL	Employment status of household head	Nominal: 0-Unemployed, 1-Otherwise	−/+
HH	Household size	Ordinal: 0-Less than 3, 1-Otherwise	+
GEN	Gender of household head	Nominal: 0-Male, 1-Female	+
SOURCEINC	Main source of income	Nominal: 0-Formal employment, 1-Otherwise	+

The Probit model estimated the probability that a household i, with characteristics $X_{i}$ had a food system decision-maker based on the following (Eq. (10)):

(10) $$P\left(D_{i}|X_{i}\right)=\varphi X_{i}^{\prime }\beta )$$

where φ is the cumulative distribution function of the standard normal distribution. Kernel and nearest neighbor methods were used to match households which had a certain food system decision-maker to those that did not by using propensity score values for estimating the ATET. In the nearest neighbor matching method, for household which had a certain food system decision-maker i and households that did not, i−1, the absolute difference between the propensity scores was as follows (Eq. (11)):

(11) $$\left|{\mathrm{Pr}}_{i}-{\mathrm{Pr}}_{i-1}\right|=\min_{k\in I=0}\left\{{\mathrm{Pr}}_{i}-{\mathrm{Pr}}_{k}\right\}$$

The non-parametric kernel matching method compared each household that had a certain food system decision-maker to a weighted average of outcomes of all household that did not, placing higher weights to those that did not with propensity scores closer to that which had a certain food system decision-maker. In the Kernel matching method, for an intrahousehold food system decision-maker i, the associated matching outcome was as follows (Eq. (12)):

(12) $$\hat{Y_{i}}=\frac{\sum_{i-1\epsilon I=0}K\left(\frac{{\mathrm{Pr}}_{i}-{\mathrm{Pr}}_{i-1}}{h}\right)Y_{i}}{\sum_{i-1\epsilon I=0}K\left(\frac{{\mathrm{Pr}}_{i}-{\mathrm{Pr}}_{i-1}}{h}\right)Y_{i}}$$

where K(·) is a kernel function, and h is a bandwidth parameter.

iii. Monte Carlo Simulation

Monte Carlo Simulations (MCS) evaluated the deterministic poison distribution, using a set of random numbers as inputs [56]. The uncertainty was the intrahousehold food system agency (i.e., number of decisions made). A Monte Carlo Filtering (MCF) method as that used by Ngarava [56] and Mary, Phimister and Roberts [57] was used in identifying the most influential parameters affecting the outcomes (i.e. the number of decisions made). The general MSC model shows that it evaluates an integral [58,59] (Eq. (13))

(13) δ=Eα{U(X)}=∫U(x)α(x)dx

where Eα{} is the expectation with respect to the probability density α and X={μ,β} is a vector of decision parameters, μ, (number of household food system decisions) and a combination, β, of system parameters (age, educational levels, tenure, employments status, household size and gender) and state variables calculated by the models. The symbol U() denotes a response function in this case the number of intrahousehold decision-making variability. The MCS evaluates the integral by generating random draws $X=x^{m}$ from the target distribution α and then estimating δ as the average of $U(x^{1}\ldots \text{,}U\left(k\right)$, where k is the number of replications, which was 100 000 in this case.

As alluded to earlier, β is a combination of system parameters (i.e., the state of nature), $\beta_{0}$, and state variables $\beta_{0}=\left\{\beta_{0},\ldots ,\beta_{Ts}\right\}$ calculated by the model. This splitting produces the following reformulation (Eq. (14)):

(14) $$\delta =E_{\alpha_{0}}\left\{E_{\alpha_{s|0}}\left\{U\left(X\right)\right\}\right\}=\int \left\{\int U\left(x\right)\frac{\alpha \left(x\right)}{\alpha_{0}\left(\beta_{0}\right)}d\left\{\mu ,\beta_{s}\right\}\right\}\mu_{0}\left(\beta_{0}\right)d\beta_{0}$$

where $E_{\alpha_{0}}$ {} is the expectation with respect to the probability density $\alpha_{0}$ of the state of nature and $E_{\alpha_{s|0}}$ {} is the expectation with respect to the conditional probability density given a state of nature. The double integral may be numerically evaluated through drawing n states of nature, $\beta_{0}^{1},\ldots ,\beta_{n}^{n}$, at random from the distribution $\alpha_{0}$. A random sample for each b observation from the distribution $\alpha_{s|0}$ is created by the simulations d times. Thus the total number of simulations run equals b=n×d [60]. STATA 14 and SPSS 27 were used to analyze the data. Data used in the study was collected through questionnaire observing ethical issues such as anonymity, confidentiality and integrity with informed consent (Ethical Clearance No: NWU-01216-21-S3 Law)

## 4 Results

### 4.1 Descriptive statistics

The average age of the respondents across the 3 study sites was more than 48 years, with family sizes ranging between 4 and 5 (Table 4). There has been generational knowledge within the study sites with households having established themselves between 23 and 43 years. The average monthly income of the respondents ranged between R2 731.39 and R3 301.47, with income proportion of food also varying between 44.54 % and 50.32 %.

**Table 4:** Descriptive statistics.

		Municipality		
		Matatiele (n = 549)	Greater Taung (n = 556)	Magareng (n = 79)
House head age (years)	Mean	55,49	57,33	48,76
	Min	18	18	21
	Max	103	98	94
Household size	Mean	5,05	4,48	3,62
	Min	1	1	1
	Max	19	19	11
Duration of stay (years)	Mean	39,97	43,03	22,95
	Min	1	1	1
	Max	200	200	94
Total monthly household income (Rand)	Mean	3301,47	2887,08	2731,39
	Min	–	–	–
	Max	36000	30000	12000
Food expenditure (Rand)	Mean	1410,72	1202,88	1117,09
	Min	50	–	–
	Max	5000	5000	3000
Income proportion of food expenditure	Mean	48,25 %	50,32 %	44,54 %
	Min	2,04 %	–	–
	Max	100 %	100 %	100 %
			**%**	
Household head gender	Male	50,27	40,83	53,16
	Female	49,73	58,63	46,84
Household head ethnicity	Afrikaner	–	–	7,59
	Colored	–	0,18	5,06
	Tswana	–	91,73	77,22
	Tsonga	–	0,36	–
	Venda	–	0,18	–
	White	0,18	–	–
	Xhosa	53,92	4,14	3,80
	Zulu	7,83	1,08	2,53
	Sotho	38,07	2,34	1,27
	Pedi	–	–	2,53
Household head marital status	Single	25,32	44,96	54,43
	Married (monogamous)	42,81	21,76	12,66
	Married (polygamous)	2,37	5,40	6,33
	Widow	13,30	12,77	6,33
	Widower	5,28	6,29	–
	Divorced	0,73	2,16	1,27
	Separated	3,46	1,26	2,53
	Living with partner	6,74	5,40	16,46
Household head highest educational level	None	3,10	12,95	17,72
	Pre-school	4,19	2,52	11,39
	Primary	51,91	30,94	32,91
	Secondary	35,88	49,10	29,11
	Tertiary	4,92	4,50	8,86
Household head employment status	Unemployed	67,76	83,09	59,49
	Formal employment in non-agricultural related activities	8,01	8,09	11,39
	Formal employment in agricultural related activities	3,83	2,16	18,99
	Informal/self-employment in non-agricultural related activities	8,20	5,40	2,53
	Informal/self-employment in agricultural related activities	12,20	1,26	7,59
Main source of income	Formal employment in non-agricultural related activities	10,20	8,99	11,39
	Formal employment in agricultural related activities	4,19	3,06	18,99
	Informa/self-employment in non-agricultural related activities	7,83	5,40	–
	Informal/self-employment in agricultural related activities	6,19	0,72	8,86
	Social grant/Pensioner	67,76	72,30	54,43
	Remittances	0,55	2,34	–
	Other	3,28	7,19	6,33

There were more female than male household heads in Matatiele (50.2 %) and Magareng (53.16 %), relative to Greater Taung (58.63 %), which had more male house heads. Most of the household heads were of Tswana ethnic group in Greater Taung (91.73 %) and Magareng (77.22 %), while in Matatiele they were Xhosa (53.92 %). Most of the household heads from the 3 study sites were either single or married in monogamous unions. Most of the household heads had either primary or secondary education, unemployed and had social grants/pensions as main source of income from the study sites.

### 4.2 Who makes intrahousehold food system decisions vis-à-vis their food security status?

**Fig. 3:**
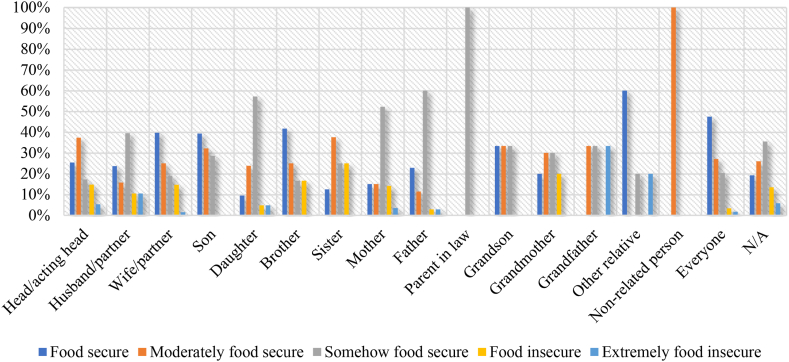
Food security status and household crop and vegetable production decision-makers.

Fig. 3 indicates that within the households, when wife (39.71 %), son (39.29 %), brother (25.00 %) and other relatives (60.00 %) make crop and vegetable production decision, there are higher incidences of food security. This is relative to instances when husband (10.53 %), daughter (4.76 %) and mother (3.54 %) make such decisions, resulting in instances of extreme food insecurity.

**Fig. 4:**
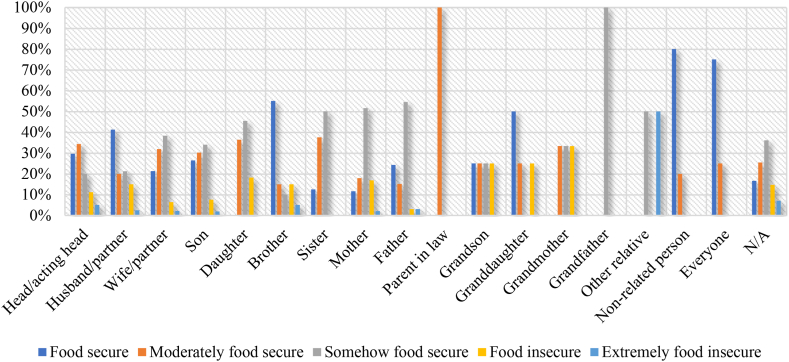
Food security status and household livestock production decision-makers.

In instances where other non-related persons (80.00 %) and everyone (75.00 %), including husband (41.25 %), brother (55.00 %) and granddaughter (50.00 %) make livestock production decisions, there is high food security (Fig. 4). Surprisingly, extreme food insecurity is realized when brother (5.00 %), father (3.03 %) and husband (2.50 %) make livestock production decisions.

**Fig. 5:**
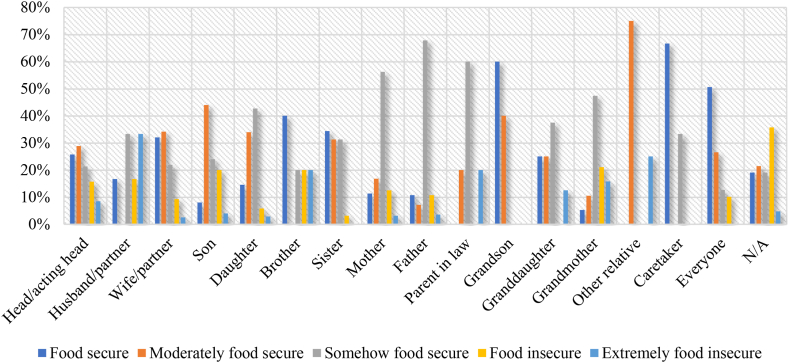
Food security status and household cooking decision-makers.

There are higher instances of food security when the caretaker (66.67 %) and everyone (50.63 %), including brother (40.00 %), sister (34.38 %) and grandson (60.00 %) make household food cooking decisions (Fig. 5). Higher incidences of food insecurity occur when husband (33.33 %), other relative (25.00 %), brother (20.00 %) and parent-in-law (20.00 %) make household cooking decisions.

**Fig. 6:**
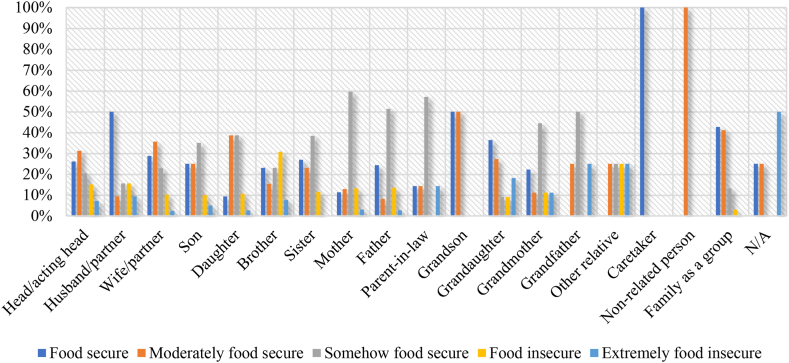
Food security status and household food expenditure decision-makers.

In terms of food expenditure, food security is mostly realized when everyone (42.65 %) including husband (50.00 %), grandson (50.00 %), granddaughter (36.36 %) and caretaker (100.00 %) make the decisions (Fig. 6). Extreme food insecurity occurs in instances where food expenditure decisions are made by other relatives (25.00 %), grandfather (25.00 %), granddaughter (18.18 %) and parent-in-law (14.29 %).

### 4.3 Determinants of intrahousehold food system agency

Table 5 shows the determinants of intrahousehold food system agency. The overall models for female-head and other none-nuclear relative making food system decisions were significant at 5 % level, respectively. Hence, further analysis will only concentrate on these models. Factors such as duration of stay, tenure, employment status and household size were significant determinants for the number of intrahousehold food system decisions made by female heads. In households where the food system decisions were made by other none-nuclear family members, factors such as location, age, gender, educational level, duration of stay, tenure, employment status and household size were significant.

**Table 5:** Poisson regression of determinants of intrahousehold food decision-making.

	Household food system decision-maker				
Variable	Male head	Female head	Son	Daughter	Other
Location	−0.003 (0.04)	−0.07 (0.05)	0.004 (0.04)	−0.03 (0.04)	0.18*** (0.06)
Age	−0.0006 (0.001)	−0.001 (0.001)	−0.0006 (0.001)	−0.002 (0.001)	0.01*** (0.001)
Gender	0.09*** (0.03)	0.01 (0.04)	0.005 (0.03)	−0.01 (0.03)	−0.19*** (0.05)
Ethnicity	0.0003 (0.009)	0.004 (0.01)	−0.002 (0.01)	−0.0008 (0.009)	−0.002 (0.01)
Marital status	−0.001 (0.008)	−0.0008 (0.01)	0.0006 (0.008)	0.002 (0.008)	−0.007 (0.01)
Educational level	−0.008 (0.02)	−0.02 (0.02)	0.005 (0.02)	−0.006 (0.02)	0.06** (0.03)
Duration of stay	0.0002 (0.0006)	0.002*** (0.0006)	−0.000 (0.0006)	−0.000 (0.0006)	−0.004*** (0.001)
Tenure	0.02 (0.02)	0.11*** (0.03)	−0.008 (0.02)	−0.008 (0.02)	−0.28*** (0.05)
Employment status	−0.01 (0.01)	−0.04** (0.02)	−0.001 (0.01)	0.001 (0.01)	0.10*** (0.02)
Household size	−0.003 (0.006)	−0.02*** (0.007)	−0.0004 (0.006)	−0.006 (0.006)	0.05*** (0.01)
Total monthly income	0.000 (0.000)	0.000 (0.000)	−0.000 (0.000)	0.000 (0.000)	−0.000 (0.000)
Food proportion of income	−0.000 (0.0005)	0.003 (0.0004)	0.000 (0.0004)	0.000 (0.0004)	−0.001 (0.001)
Constant	1.33*** (0.13)	1.17*** (0.14)	1.40*** (0.12)	1.51*** (0.13)	−0.14 (0.19)
**Summary statistics**					
Chi-square	0.63	53.17***	1.08	5.35	173.53***
Pseudo R^2^	0.003	0.014	0.000	0.002	0.048

Standard Error in parentheses.

Sig at ***1 %, **5 % and *10 %.

Table 4 shows that as the duration of stay within the communities increase, female heads where likely to make less intrahousehold food systems decisions. However, when the household size increased, they were likely to make more decisions. In terms of tenure, female heads were likely to exhibit more intrahousehold food system agency when the household owned their place of residence. The table further shows that there was less intrahousehold food system decision-making exhibited by female heads in households where the household head was unemployed.

Table 5 also shows that there are varied number of intrahousehold food system decisions made by other none-nuclear family members based on the location, with Matatiele having the highest and Magareng having the lowest. The younger the household head, the higher the number of intrahousehold food system decisions made by none-nuclear family members. When the household head gender is female, the higher the number of intrahousehold food systems decisions made by none-nuclear family members. In addition, the lower the education of the household head, the higher the agency of none-nuclear family members on intrahousehold food system decision-making. Also, the lower the duration of stay of the household within the community the lower the number of intrahousehold food system decision-making by none-nuclear family members. Further, when the household own their dwelling in terms of tenure, the less the intrahousehold food system agency by none-nuclear family members. There is more agency by none-nuclear family members in intrahousehold food system decision-making when the household head is unemployed as well as for smaller household sizes.

### 4.4 Impact of intrahousehold food system agency on decision-making and food security status

Table 6 shows that for both female-head as well as other none-nuclear family member intrahousehold food system decision-making, the overall number of decisions will decrease based on the impact of decision-maker. However, there are differing impacts on food security where households with female heads who make decision concerning crop, vegetable and livestock production decisions, the HFIAS decreases by 5 %, while those that have food expenditure decisions, HFIAS decreases by 6 %. Unlike other none-nuclear family decision-makers where HFIAS increases by 5 % when they make cooking and food expenditure decisions for the household while it increases by 4 % when they make crop and vegetable production decisions.

**Table 6:** Impact of female-head and other none-nuclear family member agency on household food system decision-making and food security.

	Household food system decision-maker														
Decision	Female head						Other								
	No. of food system decisions	HFIAS		Matching			No. of food system decisions			HFIAS			Matching		
			Kernel	% bias	Nearest neighbor	% bias					Kernel	% bias		Nearest neighbor	% bias
Crop and vegetable production	−2.40*** (0.09)	−0.05** (0.02)	−0.03	0.02	−0.02	−0.07	−2.34*** (0.07)		0.04*** (0.02)		0.03	−0.004		0.02	0.01
Livestock production	−2.27*** (0.10)	−0.05*** (0.02)	−0.05	−0.004	−0.04	0.006	−2.22*** (0.06)		0.02 (0.02)		0.01	−0.01		−0.04	0.02
Cooking	−2.19*** (0.05)	−0.05*** (0.01)	−0.03	−0.007	−0.02	−0.007	−2.32*** (0.05)		0.05*** (0.02)		0.04	0.000		0.04	0.000
Food expenditure	−2.14*** (0.05)	−0.06*** (0.01)	−0.04	−0.02	−0.00	−0.02	−2.22*** (0.06)		0.05*** (0.02)		0.05	0.004		0.07	−0.02
Kernel density diagnostic plots															

Standard Error in parentheses.

Sig. at ***1 % and **5 %.

### 4.5 Effects of uncertainty on intrahousehold food system agency

**Fig. 7:**
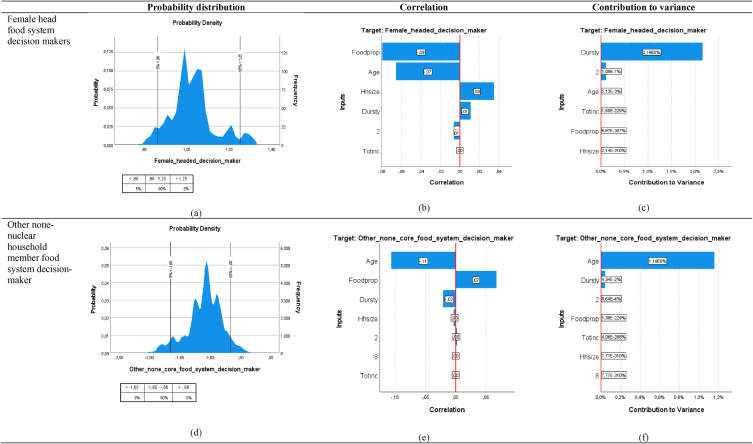
Monte Carlo Simulation results (at 100 000 replications) for household food system decision-maker (female-headed and none-nuclear family member).

Simulation in intrahousehold characteristics through varying the duration of stay and household size by ±10 %, as well as changing tenure and employment status resulted in a 90 % probability of the number of intrahousehold decisions made by female-heads decreasing (Fig. 7a). However, if these are varied for other none-nuclear family members, the number of decisions will likely increase (Fig. 7d). Income proportion on food had the highest correlation with female-head decision making (Fig. 7b), while age of household head had highest correlation with none-nuclear family member decision-making (Fig. 7e). Duration of stay within an area contributed the largest to variance in the number of decisions made by female-heads, while age contributed the largest for other none-nuclear household member decision making (Fig. 7c and f). Thus, for female head decision-making, the longer the household has been in existence within that community, the more likely that will affect the number of intrahousehold food system decisions made by female household head. Equally also, the variations in the age of the household head will also trigger a change in the number of intrahousehold food system decisions made by other none-nuclear household members.

## 5 Discussion

The results showed that there was increased female head intrahousehold food system agency in households that were bigger and owned their place of residence, having stayed in the community for a relatively short period with reduced agency when the household head was unemployed. The results also showed that there was increased intrahousehold food system agency by none-nuclear family members for household heads that were in different locations (Matatiele), relatively younger, none-owners of their place of residence, female headed, lowly educated, unemployed, had small household sizes and stayed in the community for a relatively longer period.

Monterrosa et al. [61] attest that there are intrahousehold food task differentials shaped by class and income where high income households utilize females to acquire and cook food. Female land ownership as promoted through the White Paper on South African Land Policy of 1997 has encouraged their participation in self-sustaining agricultural activities increasing their agency in food production [62]. The country's Water Research Commission has also been actively involved in promoting female food production through home gardening which has improved their agency [63]. Social protection policies such as child grant have also been instrumental in women empowerment and promoting household food security [64]. It is usually the females that receive the grants on behalf of their dependents and also decide on the food purchases. However, this also presents room for none-nuclear family members to decide on intrahousehold food systems when they receive the grants on behalf of their extended family. In South Africa, Ngarava et al. [65] found that it is females who were responsible for food preparations, purchase and home gardening. Household head unemployment determines the income levels in the house, thereby affecting female head agency within the house. Social protection policies such as child grant have been instrumental in abating food insecurity for unemployed households. It is also mostly female members of the households that receive the grant on behalf of the beneficiaries, and also exercise agency when they make food purchase decisions using the grant [64]. This has been augmented by other social protection policies such as indigent support policy [28] which provides subsidized water and electricity, increasing disposable income which is used for food purchases. The mere fact that indigency is most prevalent amongst women, who suffer mostly from water and energy insecurity [66,67], the policy has indirect benefits for their food security. This can also explain the increase of other none-nuclear family member agency in intrahousehold food system decision making for unemployed household heads.

A short duration of stay does not fully establish male dominance linked to gender norms which can be shaped by the community [68]. However, when there is longer stay within communities, there are increased possibilities of intergenerational transfers and decision-making, allowing none-nuclear household members to increase their agency in intrahousehold food system decision-making [69]. In addition, Bernard et al. [18] indicates that there is typical intrahousehold decision-making determined by community norms relative to individual preferences. The longer the household stays within a particular community, the bigger the influence of the norms in intrahousehold decision-making shaped by extended none-nuclear family decision-makers.

Bertocchi, Brunetti and Torricelli [70] indicated that household decision-making responsibility is associated with household size. Household size affects allocation patterns as well as individual intake, consequent also to entitlements established through land ownership (tenure) and rights [23]. According to Gupta, Ksoll and Maertens [71], extended family, through social norms, normally make decisions, especially older males sometimes in discussion with other older males. They are involved in the tenure decisions as well as food sharing, tending to influence intrahousehold food systems. This is complemented by patrilineal culture of women reproductive capacity and son-bearing amongst extended families, affecting decision-making even amongst women themselves [72]. This can also induce free riding in the instance where food inflows exceed outflows [71]. However, empowerment policies such as land reform and child grant have actively encouraged female participation in food production and purchase increasing their agency [64,73].

Educational levels have a bearing on intrahousehold bargaining positions [5]. Low education tends to reduce the power thresholds within households [74]. This is augmented by the relatively low paying employment accompanying low educational levels as well as adoption of convectional practices having positive effect on their food production capacity. This provides leeway for extended family members to increase their intrahousehold food system agency by affecting various aspects of food production, purchase and consumption. In terms of age, De Backer, Holvoet and Milanzi [19] indicated that it is associated with wisdom and life experience, thereby holding a bearing in households were the nuclear-family were relatively young to the extended family members. This increased intrahousehold food system agency exhibited by the external family. The South African government has been actively encouraging improved access to education. This is through policies such as accommodating students who are unable to pay their fees in public schools. Some educational institutions have also been classified as “no fee schools” with some requiring exemption to pay fees [75]. This has gone a long way in improving the educational levels and ultimately intrahousehold agency.

The results showed that based on the intrahousehold food system decision made (i.e., vegetable, crop and livestock production, as well as food expenditure), female head and other none-nuclear family member agency decreased. However, female head agency in intrahousehold food system increased food security whilst it decreased it for none-nuclear family members. In essence, even though females tend to make fewer intrahousehold food system decisions, their decisions tend to have a positive impact on food security, whilst it was opposite for none-core family members who decreased food security. Duration of stay in the community had the highest contribution to variation in agency for both female head and none-nuclear family member intrahousehold food system decision-making. Sariyev et al. [76] had findings of women agency improving food security. This was augmented by Ngarava et al. [65] highlighting differentiated intrahousehold food utilization mostly dominated by females and their roles in food production, purchase and preparation in South Africa. In Mali, Guirkinger, Platteau and Goetghebuer [77] found that there was lower agricultural productive efficiency, which tends to affect food security for collective families systems compared to female individual ones. Furthermore, there is adoption of less labor-intensive agricultural productive technologies in extended families and decision-makers compared to nuclear ones [71,78]. Intrahousehold efficiency in decision-making can be undermined by extended-family social norms of imposing patterns of behavior, thereby conflicting with efficiency and affecting food security. Furthermore, there is unequal access of food due to unequal status from social hierarchies in extended families [23,71,79,80].

## 6 Conclusion and recommendation

In conclusion, different intrahousehold members have varied intrahousehold food system agency based on the utility obtained. This is in the alley of Sen [44,45] capability approach evaluating individual ability to exercise agency, based on their economic, social, personal, cultural and institutional capacities allowing them to do so. The functionings, in this case are the various freedoms in choosing intra-households food system decisions to partake in while the functions relate to the welfare outcomes, which are the number of decisions made (utility) and food security obtained. This is augmented by the differing bargaining power the household (and non-household) members have. Furthermore, variations in the socio-economic factors of the households results in varied agency, especially for female household heads and other none-nuclear family members. Thus, the status quo is not permanent, allowing for variations in decision-making based on developing circumstances e.g., the birth of son, the death of a husband and welcoming of an in-law, etc.

Female head intrahousehold food system agency had positive impact on food security fostered by bigger households, who had stayed in the community for a relatively short time and owned their place of residence. None-nuclear family household agency had negative impact on food security which was augmented by the differential locations, and household heads who were young, uneducated, unemployed, female-headed, small size who were non-owners of their residential places and had stayed in the community for a long time. Thus, reproductive capacity (affecting household size), tenure and patrilineal generational culture play major parts in intrahousehold food system agency. There is need to promote intrahousehold food system agency by female heads in lieu of increase in food security despite their reduced decision-making. Cash transfer programmes can be conduits to target and improve female head intrahousehold food system agency. However, this is not so clear-cut in none-core family agency. To capture the real variations in intrahousehold food system agency, there is need for future research that takes a long-term panel design, due to the ever-changing family structures.

## Limitations

The study was spatially limited to Matatiele, Magareng and Greater Taung Local Municipalities, and temporally to a cross sectional survey. External validity was therefore limited, and any findings cannot cut across the whole of South Africa, spatially and temporally. This is due to heterogeneity in South Africa; therefore, the findings can only be cautiously implemented across the country. This was partially reduced by the Monte Carlo Simulations which had 100 000 iterations. Internal validity was also limited especially in measuring intra-household agency. There is heterogeneity in agency based on age differences especially for nuclear family members who are not household heads. There is an inverse relationship between age and intra-household agency. The lower the age, the lower the intra-household food system agency for nuclear family members. This was not captured in the study. Intra-household food system agency also goes beyond production, cooking and expenditure. Just like macro level food systems, intra-households’ agency also involves other aspects such as transportation and consumption decisions. This is an area that further studies can pursue.

## Data availability

The authors do not have permission to share data.

## CRediT authorship contribution statement

**Saul Ngarava:** Conceptualization, Formal analysis, Investigation, Methodology, Writing – original draft, Writing – review & editing.

## Declaration of competing interest

The authors declare that they have no known competing financial interests or personal relationships that could have appeared to influence the work reported in this paper.
