# Supplementary material for: Takes more than two to tango: Intrahousehold food system agency and its intricacies in South Africa
Source: Heliyon. 2023 Nov 10;9(11):e21770. doi: 10.1016/j.heliyon.2023.e21770 (PMC10661508; doi:10.1016/j.heliyon.2023.e21770)
Supplement: Multimedia component 1 [file mmc1.pdf]

Matatiele - Varlhaarts WEF Nexus governance for Social Justice

I am conducting research on the Water-Energy-Food Nexus governance for social justice. It focusses on the policies, laws and decision-making concerning the distribution and use of water, energy and food resources. The research is a collaboration between University of Fort Hare, North West University, University of Utrecht and University of Groningen. I seek permission (through the Consent Form) to interview you for through this questionnaire. The identification of household included in the study was completely random guided by the level of water, energy and food security through liaison with the Local Municipality and Tribal Authorities (Chiefs). Participation is completely voluntary, anonymous and you can withdraw or decline to participate at any time. The questionnaire targets information and decision making by the household head, absence of which any adult aged 18+ can answer on behalf of the household head. If you have any questions about this questionnaire, please contact the Chair of the Research Ethics Committee (LAW-Rec) of the North-West University, Dr Abraham Klaasen, Faculty of Law Tel: 018 299 2125; Fax: 018 2991923; Email: Braam.Klaasen@nwu.ac.za. Thank you for taking the time to complete in this questionnaire jointly with me. It should take 60 minutes of your time.

Name of interviewer

Date of interview

yyyy-mm-dd hh:mm

District

Municipality

Ward

Suburb/Village

HOUSEHOLD SOCIO-DEMOGRAPHIC AND ECONOMIC INFORMATION

Age of respondent

Household head age (same as before if respondent is household head)

Gender of respondent

- ◯ Male
- ◯ Female
- ◯ Other

Household head gender (same as before if respondent is household head)

- ◯ Male
- ◯ Felame
- ◯ Other

Role of respondent in the household

- ◯ Head/acting head
- ◯ Husband/partner
- ◯ Wife/partner
- ◯ Son
- ◯ Daughter
- ◯ Brother
- ◯ Sister
- ◯ Mother
- ◯ Father
- ◯ Parent-in-law
- ◯ Grandson
- ◯ Granddaughter
- ◯ Grandmother
- ◯ Grandfather
- ◯ Other relative
- ◯ Caretaker
- ◯ Non-related person

Ethnicity of household head

- ◯ Afrikaner
- ◯ Boer
- ◯ Coloured
- ◯ Tswana
- ◯ Tsonga
- ◯ Venda
- ◯ White
- ◯ Xhosa
- ◯ Zulu
- ◯ Ndebele
- ◯ Sotho
- ◯ Pedi
- ◯ Non-South African

Household head marital status

- ◯ Single
- ◯ Married (monogamous)
- ◯ Married (polygomous)
- ◯ Widow
- ◯ Widower
- ◯ Divorced
- ◯ Separated
- ◯ Living with partner

What is the highest level of education the household head has completed?

- ◯ None
- ◯ Pre-School
- ◯ Primary
- ◯ Secondary
- ◯ Tertiary

How many years has the household head been living in this ward/area

____

What is the tenure of household

- ◯ Own
- ◯ Rent
- ◯ Family trust

Employment status of household head

- ◯ Unemployed
- ◯ Formal employment in non-agricultural related activities
- ◯ Formal employment in agricultural related activities
- ◯ Informal/self employment in non-agricultural related activities
- ◯ Informal/self employment in agricultural related activities

What is the number of male household members?

____

What is the number of female household members?

____

Main source of income

- ◯ Formal employment in non-agricultural related activities
- ◯ Formal employment in agricultural related activities
- ◯ Informal/self employment in non-agricultural related activities
- ◯ Informal/self employment in agricultural related activities
- ◯ Social grant/Pensioner
- ◯ Remittances
- ◯ Other

Is there a credit or microfinance group in your area?

- ◯ Yes
- ◯ No
- ◯ I don't know

Who in your household is part of a credit or microfinance group? [more than 1 answer permitted]

- ☐ Head/acting head
- ☐ Husband/partner
- ☐ Wife/partner
- ☐ Son
- ☐ Daughter
- ☐ Brother
- ☐ Sister
- ☐ Mother
- ☐ Father
- ☐ Parent-in-law
- ☐ Grandson
- ☐ Granddaughter
- ☐ Grandmother
- ☐ Grandfather
- ☐ Other relative
- ☐ Caretaker
- ☐ Non-related person
- ☐ No one

How many males have income from formal employment in non-agricultural related activities?

____

How many females have income from formal employment in non-agricultural related activities?

____

How many males have income from formal employment in agricultural related activities?

____

How many females have income from formal employment in agricultural related activities?

____

How many males have income from informal/self employment in non-agricultural related activities?

____

How many females have income from informal/self employment in non-agricultural related activities?

____

How many males have income from informal/self employment in agricultural related activities?

____

How many females have income from informal/self employment in agricultural related activities?

____

How many males have income from social grant?

____

How many females have income from social grant?

____

What is the total monthly household income from all sources in Rand? (Estimate)

____

How much of your income are you spending on water (in Rand)

____

How much of your income are you spending on energy (in Rand)

____

How much of your income are you spending on food (in Rand)

____

Who in your household makes household food expenditure decisions?

- ◯ Head/acting head
- ◯ Husband/partner
- ◯ Wife/partner
- ◯ Son
- ◯ Daughter
- ◯ Brother
- ◯ Sister
- ◯ Mother
- ◯ Father
- ◯ Parent-in-law
- ◯ Grandson
- ◯ Granddaughter
- ◯ Grandmother
- ◯ Grandfather
- ◯ Other relative
- ◯ Caretaker
- ◯ Non-related person
- ◯ Family as a group
- ◯ N/A

Who in your household makes household water expenditure decisions?

- Head/acting head
- Husband/partner
- Wife/partner
- Son
- Daughter
- Brother
- Sister
- Mother
- Father
- Parent-in-law
- Grandson
- Granddaughter
- Grandmother
- Grandfather
- Other relative
- Caretaker
- Non-related person
- Family as a group
- N/A

Who in your household makes household energy expenditure decisions?

- Head/acting head
- Husband/partner
- Wife/partner
- Son
- Daughter
- Brother
- Sister
- Mother
- Father
- Parent-in-law
- Grandson
- Granddaughter
- Grandmother
- Grandfather
- Other relative
- Caretaker
- Non-related person
- Family as a group
- N/A

FOOD AND WATER

In the past 4 weeks...

...did you worry that your household would not have enough food?

- Never
- Rarely
- Sometimes
- Often
- Always

... were you or any household member not able to eat the kinds of foods you preferred because of a lack of resources?

- Never
- Rarely
- Sometimes
- Often
- Always

... did you or any household member have to eat a limited variety of foods due to a lack of resources?

- Never
- Rarely
- Sometimes
- Often
- Always

... did you or any household member have to eat some foods that you really did not want to eat because you didn`t have any alternatives?

- Never
- Rarely
- Sometimes
- Often
- Always

... did you or any household member have to eat a smaller meal than you felt you needed because there was not enough food?

- Never
- Rarely
- Sometimes
- Often
- Always

... did you or any household member have to eat fewer meals in a day because there was not enough food?

- Never
- Rarely
- Sometimes
- Often
- Always

... was there ever no food to eat of any kind in your household because of lack of resources to get food?

- Never
- Rarely
- Sometimes
- Often
- Always

... did you or any household member go to sleep at night hungry because there was not enough food?

- Never
- Rarely
- Sometimes
- Often
- Always

... did you or any household member go a whole day and night without eating anything because there was not enough food?

- Never
- Rarely
- Sometimes
- Often
- Always

WATER

... how frequently did you or anyone in your household worry you would not have enough water for all of your household needs?

- Never
- Rarely
- Sometimes
- Often
- Always

... how frequently has your main water source been interrupted or limited e.g. water pressure, less water than expected, river dried up?

- Never
- Rarely
- Sometimes
- Often
- Always

... how frequently have problems with water meant that clothes could not be washed?

- Never
- Rarely
- Sometimes
- Often
- Always

... how frequently have you or anyone in your household had to change schedules or plans due to problems with your water situation? Activities that may have been interrupted include caring for others, doing household chores, agricultural work, income-generating activities, sleeping, etc.

- Never
- Rarely
- Sometimes
- Often
- Always

... how frequently have you or anyone in your household had to change what was being eaten because there were problems with water. E.g., for washing foods, cooking, etc.?

- Never
- Rarely
- Sometimes
- Often
- Always

... how frequently have you or anyone in your household had to go without washing hands after dirty activities because of problems with water?

- Never
- Rarely
- Sometimes
- Often
- Always

... how frequently have you or anyone in your household had to go without washing their body because of problems with water (e.g., not enough water, dirty, unsafe)?

- Never
- Rarely
- Sometimes
- Often
- Always

... how frequently has there not been as much water to drink as you would like for you or anyone in your household?

- Never
- Rarely
- Sometimes
- Often
- Always

... how frequently did you or anyone in your household feel angry about your water situation?

- Never
- Rarely
- Sometimes
- Often
- Always

... how frequently have you or anyone in your household gone to sleep thirsty because there wasn't any water to drink?

- Never
- Rarely
- Sometimes
- Often
- Always

... how frequently has there been no useable or drinkable water whatsoever in your household?

- Never
- Rarely
- Sometimes
- Often
- Always

... how frequently have problems with water caused you or anyone in your household to feel ashamed/excluded/stigmatized?

- Never
- Rarely
- Sometimes
- Often
- Always

What is the main source of food for the household?

- ◯ Purchase
- ◯ Own production
- ◯ Gift
- ◯ Barter
- ◯ Hunting/gathering

To what extent does the household depend on growing its own crops and vegetables to feed itself?

- ◯ Not at all
- ◯ Very little
- ◯ Somewhat
- ◯ Quite a bit
- ◯ A great deal

Regarding household crop and vegetable production activities, who performs these activities?

- ◯ Head/acting head
- ◯ Husband/partner
- ◯ Wife/partner
- ◯ Son
- ◯ Daughter
- ◯ Brother
- ◯ Sister
- ◯ Mother
- ◯ Father
- ◯ Parent-in-law
- ◯ Grandson
- ◯ Granddaughter
- ◯ Grandmother
- ◯ Grandfather
- ◯ Other relative
- ◯ Caretaker
- ◯ Non-related person
- ◯ Everyone
- ◯ N/A

Regarding household crop and vegetable production activities, who makes the decisions?

- ◯ Head/acting head
- ◯ Husband/partner
- ◯ Wife/partner
- ◯ Son
- ◯ Daughter
- ◯ Brother
- ◯ Sister
- ◯ Mother
- ◯ Father
- ◯ Parent-in-law
- ◯ Grandson
- ◯ Granddaughter
- ◯ Grandmother
- ◯ Grandfather
- ◯ Other relative
- ◯ Caretaker
- ◯ Non-related person
- ◯ Everyone
- ◯ N/A

To what extent does the household depend on OWN PRODUCTION of meat, dairy and eggs to feed itself?

- ◯ Not at all
- ◯ Very little
- ◯ Somewhat
- ◯ Quite a bit
- ◯ A great deal

Who performs livestock production activities?

- Head/acting head
- Husband/partner
- Wife/partner
- Son
- Daughter
- Brother
- Sister
- Mother
- Father
- Parent-in-law
- Grandson
- Granddaughter
- Grandmother
- Grandfather
- Other relative
- Caretaker
- Non-related person
- Everyone
- N/A

Who makes livestock production decision?

- ◯ Head/acting head
- ◯ Husband/partner
- ◯ Wife/partner
- ◯ Son
- ◯ Daughter
- ◯ Brother
- ◯ Sister
- ◯ Mother
- ◯ Father
- ◯ Parent-in-law
- ◯ Grandson
- ◯ Granddaughter
- ◯ Grandmother
- ◯ Grandfather
- ◯ Other relative
- ◯ Caretaker
- ◯ Non-related person
- ◯ Everyone
- ◯ N/A

What crops does the household usually grow?

- ☐ Maize
- ☐ Potato
- ☐ Beans
- ☐ Wheat
- ☐ Rice
- ☐ Other
- ☐ None

Estimated crop land size in square metres

____

What is the tenure of the land under crop production?

- ◯ Communal/traditional
- ◯ Land reform/restitution
- ◯ Private (Own farm)
- ◯ Part owner
- ◯ Permission to occupy/other right
- ◯ Rented from someone
- ◯ Other
- ◯ N/A

What vegetables does your houseold grow?

- ☐ Cabbage
- ☐ Onion
- ☐ Spinach
- ☐ Pease
- ☐ Pumpkin
- ☐ Other
- ☐ None

Estimated vegetable production land size in square metres

____

What is the tenure of the land under vegetable production?

- ◯ Communal/traditional
- ◯ Land reform/restitution
- ◯ Private (Own farm)
- ◯ Part owner
- ◯ Permission to occupy/other right
- ◯ Rented from someone
- ◯ Other
- ◯ N/A

Number of cattle owned by household

____

Number of sheep owned by household

____

Number of goats owned by household

____

Number of chicken owned by household

____

Number of pigs owned by household

____

What is the distance to grazing land in square metres

____

What is the tenure of the land under livestock production?

- ◯ Communal/traditional
- ◯ Land reform/restitution
- ◯ Private (Own farm)
- ◯ Part owner
- ◯ Permission to occupy/other right
- ◯ Rented from someone
- ◯ Other
- ◯ N/A

Regarding household food cooking, who makes the decisions?

- ◯ Head/acting head
- ◯ Husband/partner
- ◯ Wife/partner
- ◯ Son
- ◯ Daughter
- ◯ Brother
- ◯ Sister
- ◯ Mother
- ◯ Father
- ◯ Parent-in-law
- ◯ Grandson
- ◯ Granddaughter
- ◯ Grandmother
- ◯ Grandfather
- ◯ Other relative
- ◯ Caretaker
- ◯ Non-related person
- ◯ Everyone
- ◯ N/A

If you do not engage in agricultural production, or if your productivity was reduced, please specify the reason: [More than 1 answer permitted]

- ☐ No access to land
- ☐ No access to water
- ☐ No access to energy (e.g. fuel for machinery, electricity for pumping water)
- ☐ Poor soil quality
- ☐ No money to buy seeds/plants
- ☐ No interest
- ☐ No time
- ☐ Security issues
- ☐ Other
- ☐ N/A

To improve food security, what would you require to improve agricultural production? [More than 1 answer permitted]

- [ ] Grow own food
- [ ] Small vegetable garden
- [ ] Access to grazing land
- [ ] Access to water and land
- [ ] Community gardens
- [ ] A farm/big piece of land
- [ ] Training in agricultural management practices
- [ ] Other

How much time is spent per day to fetch water for household use?_ minutes/day

____

Does your household receive any of the following services from the municipality? [More than 1 answer permitted]

- [ ] Water supply
- [ ] Sewerage
- [ ] Refuse removal
- [ ] None

Do you think that when complaints regarding water are reported, the municipality effectively deals with them?

- ○ Yes
- ○ No
- ○ N/A
- ○ Other

What is the main water source for cooking in the household?

- ◯ Piped (tap) water in house
- ◯ Piped (tap) water in yard
- ◯ Borehole water in yard
- ◯ Borehole outside yard
- ◯ Rain-water tank in yard
- ◯ Neighbor's tap
- ◯ Public/communal tap
- ◯ Water-carrier/taker
- ◯ Water from stream/river
- ◯ Water from dam/pool
- ◯ Well
- ◯ Unprotected spring
- ◯ Protected spring
- ◯ Other

What is the main water source for drinking in the household?

- ◯ Piped (tap) water in house
- ◯ Piped (tap) water in yard
- ◯ Borehole water in yard
- ◯ Borehole outside yard
- ◯ Rain-water tank in yard
- ◯ Neighbor's tap
- ◯ Public/communal tap
- ◯ Water-carrier/taker
- ◯ Water from stream/river
- ◯ Water from dam/pool
- ◯ Well
- ◯ Unprotected spring
- ◯ Protected spring
- ◯ Other

What is the main water source for bathing in the household?

- Piped (tap) water in house
- Piped (tap) water in yard
- Borehole water in yard
- Borehole outside yard
- Rain-water tank in yard
- Neighbor's tap
- Public/communal tap
- Water-carrier/taker
- Water from stream/river
- Water from dam/pool
- Well
- Unprotected spring
- Protected spring
- Other

What is the main water source for washing dishes in the household?

- Piped (tap) water in house
- Piped (tap) water in yard
- Borehole water in yard
- Borehole outside yard
- Rain-water tank in yard
- Neighbor's tap
- Public/communal tap
- Water-carrier/taker
- Water from stream/river
- Water from dam/pool
- Well
- Unprotected spring
- Protected spring
- Other

What is the main water source for washing clothes in the household?

- ◯ Piped (tap) water in house
- ◯ Piped (tap) water in yard
- ◯ Borehole water in yard
- ◯ Borehole outside yard
- ◯ Rain-water tank in yard
- ◯ Neighbor's tap
- ◯ Public/communal tap
- ◯ Water-carrier/taker
- ◯ Water from stream/river
- ◯ Water from dam/pool
- ◯ Well
- ◯ Unprotected spring
- ◯ Protected spring
- ◯ Other

What is the main water source for growing crops in the household?

- ◯ Piped (tap) water in house
- ◯ Piped (tap) water in yard
- ◯ Borehole water in yard
- ◯ Borehole outside yard
- ◯ Rain-water tank in yard
- ◯ Neighbor's tap
- ◯ Public/communal tap
- ◯ Water-carrier/taker
- ◯ Water from stream/river
- ◯ Water from dam/pool
- ◯ Well
- ◯ Unprotected spring
- ◯ Protected spring
- ◯ Other
- ◯ N/A

What is the main water source for raising livestock in the household?

- Piped (tap) water in house
- Piped (tap) water in yard
- Borehole water in yard
- Borehole outside yard
- Rain-water tank in yard
- Neighbor's tap
- Public/communal tap
- Water-carrier/taker
- Water from stream/river
- Water from dam/pool
- Well
- Unprotected spring
- Protected spring
- Other
- N/A

Who mainly uses water in household activities?

- ◯ Head/acting head
- ◯ Husband/partner
- ◯ Wife/partner
- ◯ Son
- ◯ Daughter
- ◯ Brother
- ◯ Sister
- ◯ Mother
- ◯ Father
- ◯ Parent-in-law
- ◯ Grandson
- ◯ Granddaughter
- ◯ Grandmother
- ◯ Grandfather
- ◯ Other relative
- ◯ Caretaker
- ◯ Non-related person
- ◯ Everyone
- ◯ N/A

Who decides how to use water in household activities? [more than 1 answer permitted]

- [ ] Head/acting head
- [ ] Husband/partner
- [ ] Wife/partner
- [ ] Son
- [ ] Daughter
- [ ] Brother
- [ ] Sister
- [ ] Mother
- [ ] Father
- [ ] Parent-in-law
- [ ] Grandson
- [ ] Granddaughter
- [ ] Grandmother
- [ ] Grandfather
- [ ] Other relative
- [ ] Caretaker
- [ ] Non-related person
- [ ] No one

Who fetches water for the household? [more than 1 answer permitted]

- [ ] Head/acting head
- [ ] Husband/partner
- [ ] Wife/partner
- [ ] Son
- [ ] Daughter
- [ ] Brother
- [ ] Sister
- [ ] Mother
- [ ] Father
- [ ] Parent-in-law
- [ ] Grandson
- [ ] Granddaughter
- [ ] Grandmother
- [ ] Grandfather
- [ ] Other relative
- [ ] Caretaker
- [ ] Non-related person
- [ ] No one

Who decides who fetches water for the household?[more than 1 answer permitted]

- [ ] Head/acting head
- [ ] Husband/partner
- [ ] Wife/partner
- [ ] Son
- [ ] Daughter
- [ ] Brother
- [ ] Sister
- [ ] Mother
- [ ] Father
- [ ] Parent-in-law
- [ ] Grandson
- [ ] Granddaughter
- [ ] Grandmother
- [ ] Grandfather
- [ ] Other relative
- [ ] Caretaker
- [ ] Non-related person
- [ ] No one

How is your drinking water treated [More than 1 answer permitted]

- [ ] Municipality treated
- [ ] Filtering
- [ ] Allowing to settle
- [ ] Putting chlorine
- [ ] Boiling
- [ ] Bleach
- [ ] Purifying tablets
- [ ] Other
- [ ] I don't know
- [ ] None

What are the main causes of poor water quality? [More than 1 answer permitted

- [ ] Ground water depletion
- [ ] Water pollution
- [ ] Drought
- [ ] Flooding
- [ ] Poor government service delivery
- [ ] Animal excreta close to well/spring
- [ ] People washing their bodies in a stream
- [ ] People washing clothes in a stream
- [ ] Pits/latrines close to well/spring
- [ ] Overflow drains
- [ ] Sewerage flowing into streams
- [ ] Garbage close to eell/spring
- [ ] Lack of energy
- [ ] Other
- [ ] I don't know

How frequently have you or anyone in your household felt sick after drinking water?

- ○ Not at all
- ○ Very little
- ○ Somewhat
- ○ Quite a bit
- ○ A great deal

By regularly not having access to water, or water not being clean, which of the following things are a real big problem for you? Rank in terms of problems [1 (not a problem) – 5 (real big problem)]

____

1st choice

- ○ Cooking
- ○ Drinking
- ○ Bathing
- ○ Washing clothes
- ○ Washing dishes
- ○ Growing crops
- ○ Raising livestock
- ○ Other
- ○ N/A 1
- ○ N/A 2
- ○ N/A 3

2nd choice

- Cooking
- Drinking
- Bathing
- Washing clothes
- Washing dishes
- Growing crops
- Raising livestock
- Other
- N/A 1
- N/A 2
- N/A 3

3rd choice

- Cooking
- Drinking
- Bathing
- Washing clothes
- Washing dishes
- Growing crops
- Raising livestock
- Other
- N/A 1
- N/A 2
- N/A 3

What type of toilet facility does the household have?

- Flush toilet
- Flush to septic tank
- Other, within house premises
- Communal (outside house premises)
- Pit latrine
- Bucket toilet
- Bush

Is there a latrine close to the well/spring?

- Yes
- No
- I don't know

Does the well/spring have a cover?

- Yes
- No
- I don't know

Who does the maintenance of toilet or hygiene facilities?[more than 1 answer permitted]

- [ ] Head/acting head
- [ ] Husband/partner
- [ ] Wife/partner
- [ ] Son
- [ ] Daughter
- [ ] Brother
- [ ] Sister
- [ ] Mother
- [ ] Father
- [ ] Parent-in-law
- [ ] Grandson
- [ ] Granddaughter
- [ ] Grandmother
- [ ] Grandfather
- [ ] Other relative
- [ ] Caretaker
- [ ] Non-related person
- [ ] No one

Who makes household decisions concerning maintenance of toilet or hygiene facilities?[more than 1 answer permitted]

- [ ] Head/acting head
- [ ] Husband/partner
- [ ] Wife/partner
- [ ] Son
- [ ] Daughter
- [ ] Brother
- [ ] Sister
- [ ] Mother
- [ ] Father
- [ ] Parent-in-law
- [ ] Grandson
- [ ] Granddaughter
- [ ] Grandmother
- [ ] Grandfather
- [ ] Other relative
- [ ] Caretaker
- [ ] Non-related person
- [ ] No one

ENERGY

What type of energy do you use for space heating?

- [ ] Energy from grid
- [ ] Paraffin
- [ ] Gas
- [ ] Candles
- [ ] Manure/dung
- [ ] Crop residue
- [ ] Petrol/diesel
- [ ] Fuelwood/wood
- [ ] Solar panels
- [ ] Hydropower
- [ ] Biogas
- [ ] Other
- [ ] None

What type of energy do you use for water heating?

- [ ] Energy from grid
- [ ] Paraffin
- [ ] Gas
- [ ] Candles
- [ ] Manure/dung
- [ ] Crop residue
- [ ] Petrol/diesel
- [ ] Fuelwood/wood
- [ ] Solar panels
- [ ] Hydropower
- [ ] Biogas
- [ ] Other
- [ ] None

What type of energy do you use for lighting?

- [ ] Energy from grid
- [ ] Paraffin
- [ ] Gas
- [ ] Candles
- [ ] Manure/dung
- [ ] Crop residue
- [ ] Petrol/diesel
- [ ] Fuelwood/wood
- [ ] Solar panels
- [ ] Hydropower
- [ ] Biogas
- [ ] Other
- [ ] None

What type of energy do you use for cooking?

- [ ] Energy from grid
- [ ] Paraffin
- [ ] Gas
- [ ] Candles
- [ ] Manure/dung
- [ ] Crop residue
- [ ] Petrol/diesel
- [ ] Fuelwood/wood
- [ ] Solar panels
- [ ] Hydropower
- [ ] Biogas
- [ ] Other
- [ ] None

What type of energy do you use for appliances?

- [ ] Energy from grid
- [ ] Paraffin
- [ ] Gas
- [ ] Candles
- [ ] Manure/dung
- [ ] Crop residue
- [ ] Petrol/diesel
- [ ] Fuelwood/wood
- [ ] Solar panels
- [ ] Hydropower
- [ ] Biogas
- [ ] Other
- [ ] None

What type of energy do you use for growing crops?

- [ ] Energy from grid
- [ ] Paraffin
- [ ] Gas
- [ ] Candles
- [ ] Manure/dung
- [ ] Crop residue
- [ ] Petrol/diesel
- [ ] Fuelwood/wood
- [ ] Solar panels
- [ ] Hydropower
- [ ] Biogas
- [ ] Draught power
- [ ] Other
- [ ] None

What type of energy do you use for rearing livestock?

- [ ] Energy from grid
- [ ] Paraffin
- [ ] Gas
- [ ] Candles
- [ ] Manure/dung
- [ ] Crop residue
- [ ] Petrol/diesel
- [ ] Fuelwood/wood
- [ ] Solar panels
- [ ] Hydropower
- [ ] Biogas
- [ ] Other
- [ ] None

What type of energy do you use for getting water?

- [ ] Energy from grid
- [ ] Paraffin
- [ ] Gas
- [ ] Candles
- [ ] Manure/dung
- [ ] Crop residue
- [ ] Petrol/diesel
- [ ] Fuelwood/wood
- [ ] Solar panels
- [ ] Hydropower
- [ ] Biogas
- [ ] Draught power
- [ ] Other
- [ ] None

If you are connected to the electrical grid, from who do you buy electricity from?

- ◯ Eskom
- ◯ Municipality
- ◯ I don't get electricity from the grid
- ◯ I don't know

Is the lack of electricity a frequent problem in your household?

- Yes
- No
- N/A

If yes, what do you think is the reason for the lack of electricity? [More than 1 answer permitted]

- Unreliable service provision
- Cost of electricity provision is too high for our household
- Theft of electricity lines
- Infrastructure disruption
- Other
- N/A

By regularly not having access to energy, which the following activities that are a real big problem for you? [From 1 (not a problem) – 5 (real big problem)]

1st choice

- Cooking
- Lights
- Appliances (TV, microwave, fridge, etc)
- Water heating
- Getting water
- Household heating
- Growing crops
- Raising livestock
- Other

2nd choice

- Cooking
- Lights
- Appliances (TV, microwave, fridge, etc)
- Water heating
- Getting water
- Household heating
- Growing crops
- Raising livestock
- Other

3rd choice

- Cooking
- Lights
- Appliances (TV, microwave, fridge, etc)
- Water heating
- Getting water
- Household heating
- Growing crops
- Raising livestock
- Other

How much time is spent to collecting fuelwood? minutes/day

Who collects wood fuel for energy? [more than 1 answer permitted]

- [ ] Head/acting head
- [ ] Husband/partner
- [ ] Wife/partner
- [ ] Son
- [ ] Daughter
- [ ] Brother
- [ ] Sister
- [ ] Mother
- [ ] Father
- [ ] Parent-in-law
- [ ] Grandson
- [ ] Granddaughter
- [ ] Grandmother
- [ ] Grandfather
- [ ] Other relative
- [ ] Caretaker
- [ ] Non-related person
- [ ] No one

Who makes household decisions concerning purchase or collection of fuel for energy? [more than 1 answer permitted]

- ☐ Head/acting head
- ☐ Husband/partner
- ☐ Wife/partner
- ☐ Son
- ☐ Daughter
- ☐ Brother
- ☐ Sister
- ☐ Mother
- ☐ Father
- ☐ Parent-in-law
- ☐ Grandson
- ☐ Granddaughter
- ☐ Grandmother
- ☐ Grandfather
- ☐ Other relative
- ☐ Caretaker
- ☐ Non-related person
- ☐ No one

Do you cook on a stove, indoors, using any fuel besides electricity or gas (LPG, natural gas or biogas)?

- ◯ Yes
- ◯ No

Do you cook on an open fire indoors using any fuel besides electricity or gas (LPG, natural gas or biogas)?

- ◯ Yes
- ◯ No

Who usually cooks? [more than 1 answer permitted]

- [ ] Head/acting head
- [ ] Husband/partner
- [ ] Wife/partner
- [ ] Son
- [ ] Daughter
- [ ] Brother
- [ ] Sister
- [ ] Mother
- [ ] Father
- [ ] Parent-in-law
- [ ] Grandson
- [ ] Granddaughter
- [ ] Grandmother
- [ ] Grandfather
- [ ] Other relative
- [ ] Caretaker
- [ ] Non-related person
- [ ] No one

Who makes household decisions concerning the type of cooking stove to use? [more than 1 answer permitted]

- [ ] Head/acting head
- [ ] Husband/partner
- [ ] Wife/partner
- [ ] Son
- [ ] Daughter
- [ ] Brother
- [ ] Sister
- [ ] Mother
- [ ] Father
- [ ] Parent-in-law
- [ ] Grandson
- [ ] Granddaughter
- [ ] Grandmother
- [ ] Grandfather
- [ ] Other relative
- [ ] Caretaker
- [ ] Non-related person
- [ ] No one

Does the household own any of the following? [More than 1 answer permitted]

- [ ] Refrigerator
- [ ] Radio
- [ ] Television
- [ ] Space cooling appliance
- [ ] Space heating appliance
- [ ] Landline
- [ ] Mobile phone
- [ ] Internet connection
- [ ] Personal computer
- [ ] Laptop
- [ ] Stove
- [ ] Other

Do you use the wattle/swarthaak tree as a source of energy?

- ( ) Yes
- ( ) No

If yes, how regularly do you use it?

- ◯ Everyday
- ◯ Every week
- ◯ Every month
- ◯ Over a month
- ◯ N/A

Do you use the wattle/swarthaak tree in any other way than as a source of energy?

- ◯ Yes
- ◯ No

If yes, for which activities? [More than 1 answer permitted]

- ☐ Fodder
- ☐ Fencing
- ☐ Food and medicine
- ☐ Utensils
- ☐ Other
- ☐ N/A

To what extent do you think wattle/swarthaak trees negatively affect water availability in the region?

- ◯ Not at all
- ◯ Very little
- ◯ Somewhat
- ◯ Quite a bit
- ◯ A great deal

What is your opinion on clearing the wattle/swarthaak tree?

- ◯ I am in favour
- ◯ I am against
- ◯ Do not know

GENDER, EQUITY AND INTRA-HOUSEHOLD DECISION MAKING

Who is the owner of the dwelling you reside in? [more than 1 answer permitted]

- [ ] Head/acting head
- [ ] Husband/partner
- [ ] Wife/partner
- [ ] Son
- [ ] Daughter
- [ ] Brother
- [ ] Sister
- [ ] Mother
- [ ] Father
- [ ] Parent-in-law
- [ ] Grandson
- [ ] Granddaughter
- [ ] Grandmother
- [ ] Grandfather
- [ ] Other relative
- [ ] Caretaker
- [ ] Non-related person
- [ ] No one

Are there any women in your household who own or partly own land?

- ○ Yes
- ○ No

If YES, do their names appear in the land ownership/rights in land documents/registered with the traditional authority?

- ○ Yes
- ○ No
- ○ I dont' know
- ○ N/A

GENDER, EQUITY AND GROUP/COMMUNITY DECISION MAKING

Is there an agricultural/ livestock/ fisheries producer's group (including marketing groups) in your community?

- ○ Yes
- ○ No
- ○ I don't know

Is there a water user's group in your community?

- Yes
- No
- I don't know

Is there an energy producer's group in your community?

- Yes
- No
- I don't know

Is there a ward committee in your community?

- Yes
- No
- I don't know

Is there an IDP forum in your community?

- Yes
- No
- I don't know

How effective are the agricultural, energy and water producers groups?

- Non-existent
- Very ineffective
- Ineffective
- Effective
- Very effective
- I don't know
- I don't know
- I don't know

How effective are the ward committees in water, energy and food development?

- Non-existent
- Very ineffective
- Ineffective
- Effective
- Very effective
- I don't know
- I don't know
- I don't know

How effective are the IDP forums in water, energy and food development?

- ◯ Non-existent
- ◯ Very ineffective
- ◯ Ineffective
- ◯ Effective
- ◯ Very effective
- ◯ I don't know
- ◯ I don't know
- ◯ I don't know

Who in your household is part of an agricultural an/or energy and/or water users group? [more than 1 answer permitted]

- ☐ Head/acting head
- ☐ Husband/partner
- ☐ Wife/partner
- ☐ Son
- ☐ Daughter
- ☐ Brother
- ☐ Sister
- ☐ Mother
- ☐ Father
- ☐ Parent-in-law
- ☐ Grandson
- ☐ Granddaughter
- ☐ Grandmother
- ☐ Grandfather
- ☐ Other relative
- ☐ Caretaker
- ☐ Non-related person
- ☐ No one

Who in your household is part of a ward committee and/or IDP forum? [more than 1 answer permitted]

- [ ] Head/acting head
- [ ] Husband/partner
- [ ] Wife/partner
- [ ] Son
- [ ] Daughter
- [ ] Brother
- [ ] Sister
- [ ] Mother
- [ ] Father
- [ ] Parent-in-law
- [ ] Grandson
- [ ] Granddaughter
- [ ] Grandmother
- [ ] Grandfather
- [ ] Other relative
- [ ] Caretaker
- [ ] Non-related person
- [ ] No one

In their activities, do agricultural, energy and water user's groups focus on the following? [More than 1 answer permitted]

- [ ] Gender
- [ ] Disability
- [ ] Variety of stakeholders (i.e. farmers, faith-based organizations, CBOs)
- [ ] Race
- [ ] Geographical location
- [ ] Age
- [ ] Other
- [ ] I don't know
- [ ] None
- [ ] None
- [ ] None

In their activities, do ward committees focus on the following? [More than 1 answer permitted]

- ☐ Gender
- ☐ Disability
- ☐ Variety of stakeholders (i.e. farmers, faith-based organizations, CBOs)
- ☐ Race
- ☐ Geographical location
- ☐ Age
- ☐ Other
- ☐ I don't know
- ☐ None
- ☐ None
- ☐ None

In their activities, do IDP forums focus on the following? [More than 1 answer permitted]

- ☐ Gender
- ☐ Disability
- ☐ Variety of stakeholders (i.e. farmers, faith-based organizations, CBOs)
- ☐ Race
- ☐ Geographical location
- ☐ Age
- ☐ Other
- ☐ I don't know
- ☐ None
- ☐ None
- ☐ None

Do you, through your agricultural, energy and water user groups, give recommendations regarding your water, energy or food issues?

- ○ I don't know
- ○ Never
- ○ Rarely
- ○ Regularly
- ○ N/A

Do you, through your ward committee or IDP forum give recommendations regarding your water, energy or food issues?

- I don't know
- Never
- Rarely
- Regularly
- N/A

Has any action ever been taken to improve the water, energy and food situation based on your recommendations in the agricultural/energy/water user groups/ward committees and IDP forum?

- Yes
- No
- I don't know
- N/A

Would you be willing to work together with other community members to manage the provision of water, energy and food?

- Not at all
- Very little
- Somewhat
- Quite a bit
- A great deal

Do you feel able and comfortable speaking up in public to help decide on water (like small wells, water supplies, etc.), energy (electrification, alternative energy systems, solar panel installment, etc.) and food (community land rights, agricultural practices, livestock grazing practices, etc.) related issues in your community?

- Not at all
- Very little
- Somewhat
- Quite a bit
- A great deal

Who do you think is responsible for improving water, energy and food security in your community?

- Each individual
- Traditional leaders/Chiefs
- Ward committees
- Local Municipality
- District Municipality
- Province
- National

What do you think are the reasons for failure in the implementation and upkeep of water, energy and food resources in the community? [answer as many as possible]

- [ ] Corruption
- [ ] Gender drift
- [ ] Ethnic drift
- [ ] Inequality
- [ ] Lack of cohesion
- [ ] Lack of empowerment
- [ ] Lack of right to access water, energy and food
- [ ] Lack of participation
- [ ] Other
- [ ] I don't know
- [ ] N/A

Are you aware of the relevant laws, legislation and regulations concerning water, energy and food (access, equity, diversity, participation and human rights) decisions at the community level

- ○ Not at all
- ○ Very little
- ○ Somewhat
- ○ Quite a bit
- ○ A great deal

Is community level food, energy and water decision-making adequate, accurate and timely?

- ○ Not at all
- ○ Very little
- ○ Somewhat
- ○ Quite a bit
- ○ A great deal
- ○ I don't know

Does community level food, energy and water decision-making accommodate the poor and underrepresented (gender, race, age, ethnicity)?

- ○ Not at all
- ○ Very little
- ○ Somewhat
- ○ Quite a bit
- ○ A great deal
- ○ I don't know

Is community level food, energy and water decision-making objective, independent and not influenced by individuals or groups

- ◯ Not at all
- ◯ Very little
- ◯ Somewhat
- ◯ Quite a bit
- ◯ A great deal
- ◯ I don't know

Are you aware of any indigent (pro-poor) support policies offering free energy, water and/or food

- ◯ Yes
- ◯ No
- ◯ I don't know

Which sector has an indigent (pro-poor) policy offering free energy, water and/or food that you are aware of? [Answer as many as possible]

- ☐ Water
- ☐ Energy
- ☐ Food
- ☐ N/A

Are you a beneficiary of the indigent (pro-poor) policy offering free energy, water and/or food, and if so from which sector? [Answer as many as possible]

- ☐ Water
- ☐ Energy
- ☐ Food
- ☐ N/A

If you are not a beneficiary of the indigent (pro-poor) policy offering free energy, water and/or food, what might be the reason? [More than one answer permitted]

- ☐ Household income is more than the allowable income in the indigent policy
- ☐ I am unaware of the indigent policy
- ☐ We are not connected to official water, energy and food ammenities
- ☐ Lack of resources
- ☐ Corruption
- ☐ Other
- ☐ N/A

In your view, is the indigent (pro-poor) policy offering free energy, water and/or food effective in providing water, energy and/food for the poor?

- ◯ Not at all
- ◯ Very little
- ◯ Somewhat
- ◯ Quite a bit
- ◯ A great deal

Who benefits from the implementation of the indigent (pro-poor) policy offering free energy, water and/or food in providing water, energy and food resources in the community?

- ◯ No one
- ◯ Individuals (poor/unrepresented)
- ◯ Individuals (rich/connected/politician)
- ◯ Community (poor/unrepresented)
- ◯ Community (rich/connected/politician)
- ◯ I don't know

GPS coordinates

latitude (x.y °)

longitude (x.y °)

altitude (m)

accuracy (m)

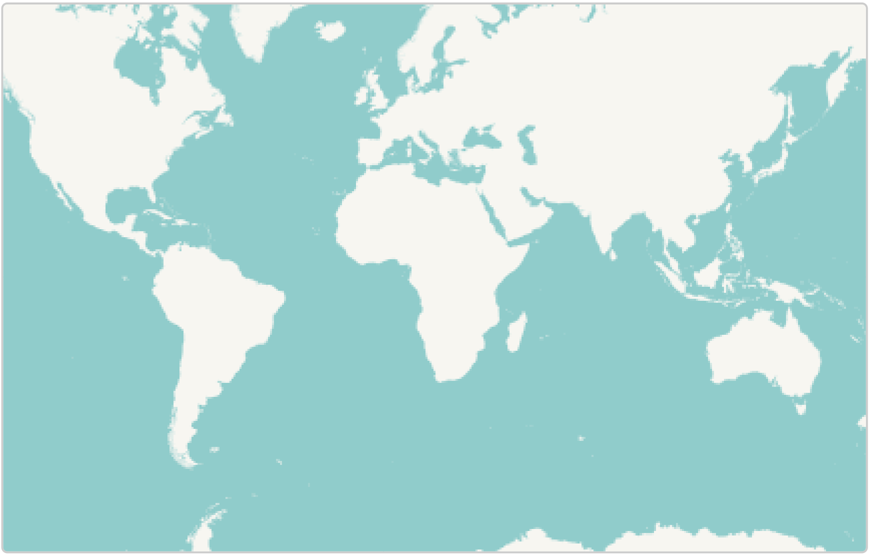

THE END
